# Supplementary material for: Optimization of the Green Chemistry-like Extraction of Phenolic Compounds from Grape (Vitis labrusca L.) and Blackberry (Rubus fruticosus L.) Seeds with Concomitant Biological and Antioxidant Activity Assessments
Source: Plants (Basel). 2023 Jul 11;12(14):2618. doi: 10.3390/plants12142618 (PMC10386244; doi:10.3390/plants12142618)
Supplement: Supplementary file 1 [file plants-12-02618-s001.zip › plants-2494263-supplementary.pdf]

Supplementary Materials

Table S1. Mass spectrometry parameters for the analysis of each phenolic compound.

**Curtain Gas (CUR)**	25 V
**Collision Gas (CAD)**	High
**IonSpray voltage (IS)**	Negative mode -4500 and positive mood 5500
**Temperature (TEM)**	400 °C
**Ion Source Gas 1 (GS1)**	55 V
**Ion Source Gas 2 (GS2)**	55 V
**Column furnace temperature**	40 °C
**Injection volume**	5 uL
**Flow**	0.3 mL/min
**0 min**	98% A
**0 to 3 min**	80 % A
**3 to 10 min**	10 % A
**10 to 11 min**	10 % A
**11 to 13 min**	98 % A
**Break-even time**	3 min
**Mobile phase A**	Water 0.1% formic acid
**Mobile phase B**	Acetonitrile 0.1% formic acid
**Column**	Zorbax Eclipse Plus C_{18} column (3.5 µm, 3.0 x 100 mm), Agilent Technologies, Inc. (Saint Clara, USA)
**LoQ**	ranged from 0.20 to 12.8 µg/L
**Range of work**	25 to 1000 ug/L
**HPLC**	1290 Infinity HPLC system from Agilent Technologies Deutschland GmbH (Waldbronn, Germany)
**Mass**	Qtrap 5500 hybrid triple quadrupole-linear ion trap mass spectrometer from AB Sciex LLC (Framingham, USA)
**Reference**	Seraglio, 2016
**Ultrapure water**	Minimum resistivity 18,3 $M\Omega cm^{-1}$
**Acetonitrile**	UV/HPLC. scientific exodus Batch: 2108034375
**Formic acid**	HoneyWell Lote #SZBG2510H

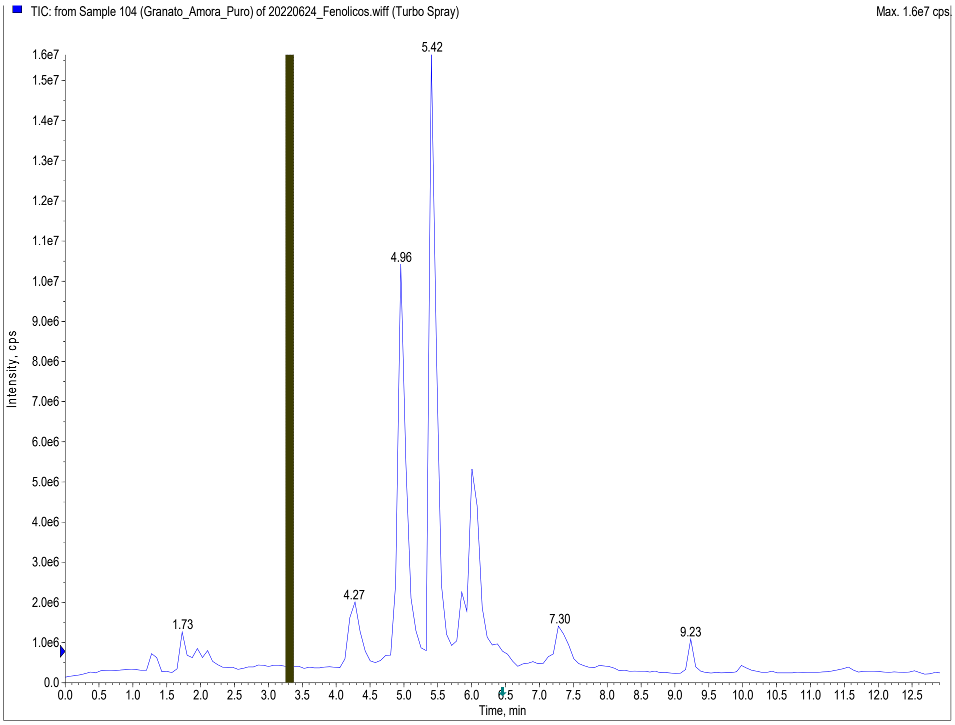
TIC: from Sample 104 (Granato_Amora_Puro) of 20220624_Fenolicos.wiff (Turbo Spray)
Max. 1.6e7 cps.
Intensity, cps
Time, min
5.42
4.96
4.27
1.73
7.30
9.23

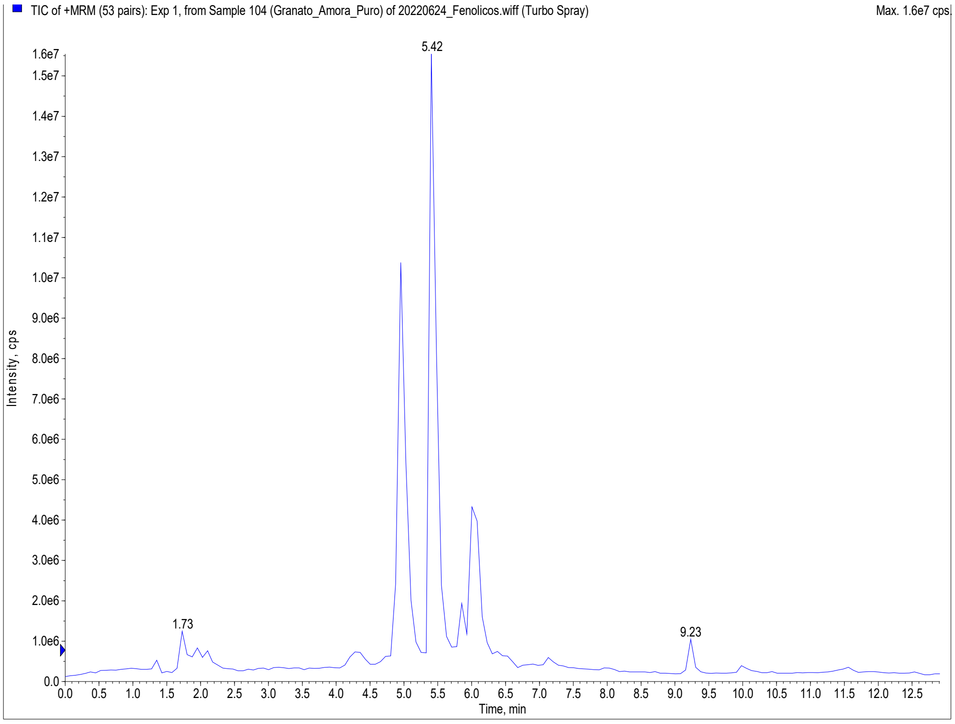
TIC of +MRM (53 pairs): Exp 1, from Sample 104 (Granato_Amora_Puro) of 20220624_Fenolicos.wiff (Turbo Spray)
Max. 1.6e7 cps.
Intensity, cps
Time, min
5.42
1.73
9.23

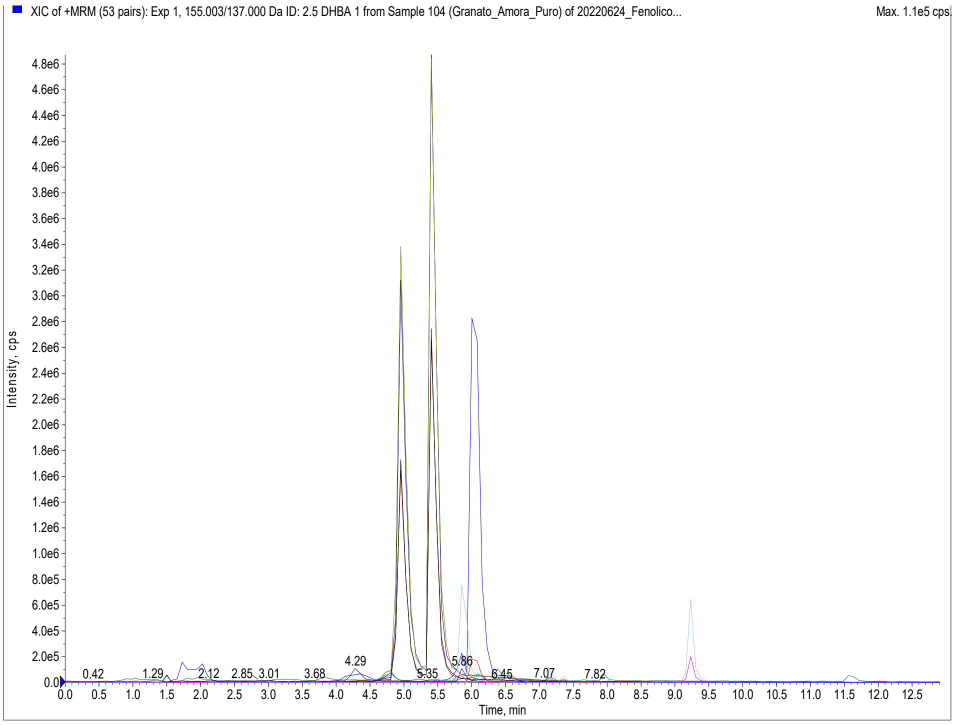
XIC of +MRM (53 pairs): Exp 1, 155.003/137.000 Da ID: 2.5 DHBA 1 from Sample 104 (Granato_Amora_Puro) of 20220624_Fenolico...
Max. 1.1e5 cps.
Intensity, cps
Time, min
0.42
1.29
2.12
2.85
3.01
3.68
4.29
5.35
5.86
6.45
7.07
7.82

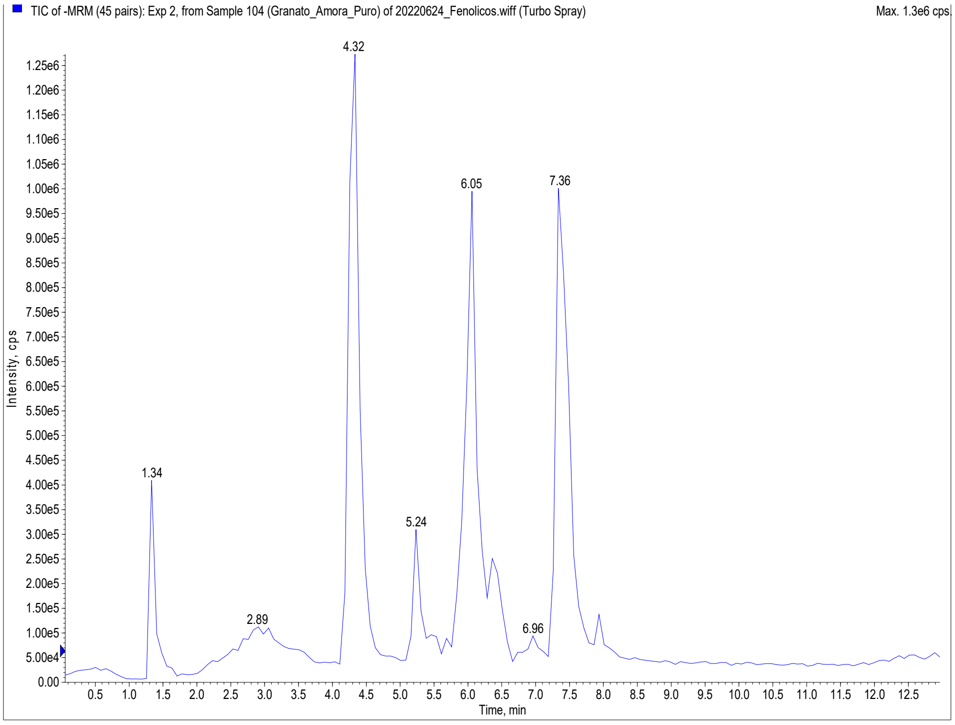
TIC of -MRM (45 pairs): Exp 2, from Sample 104 (Granato_Amora_Puro) of 20220624_Fenolicos.wiff (Turbo Spray)
Max. 1.3e6 cps.
Intensity, cps
Time, min
1.34
2.89
4.32
5.24
6.05
6.96
7.36

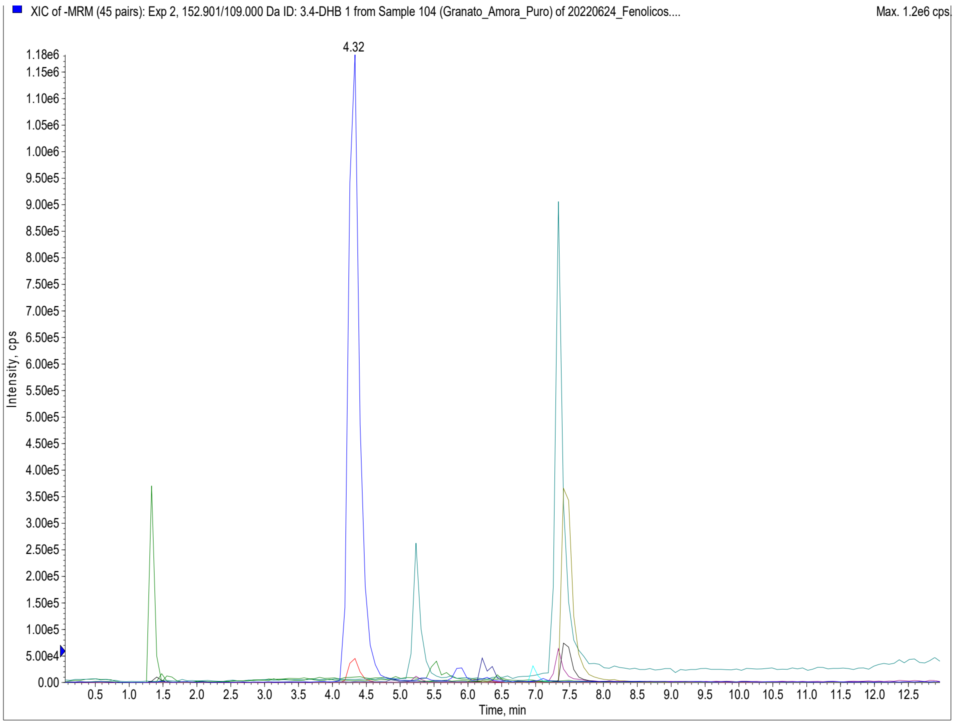

Figure S1. Blackberry chromatograms.

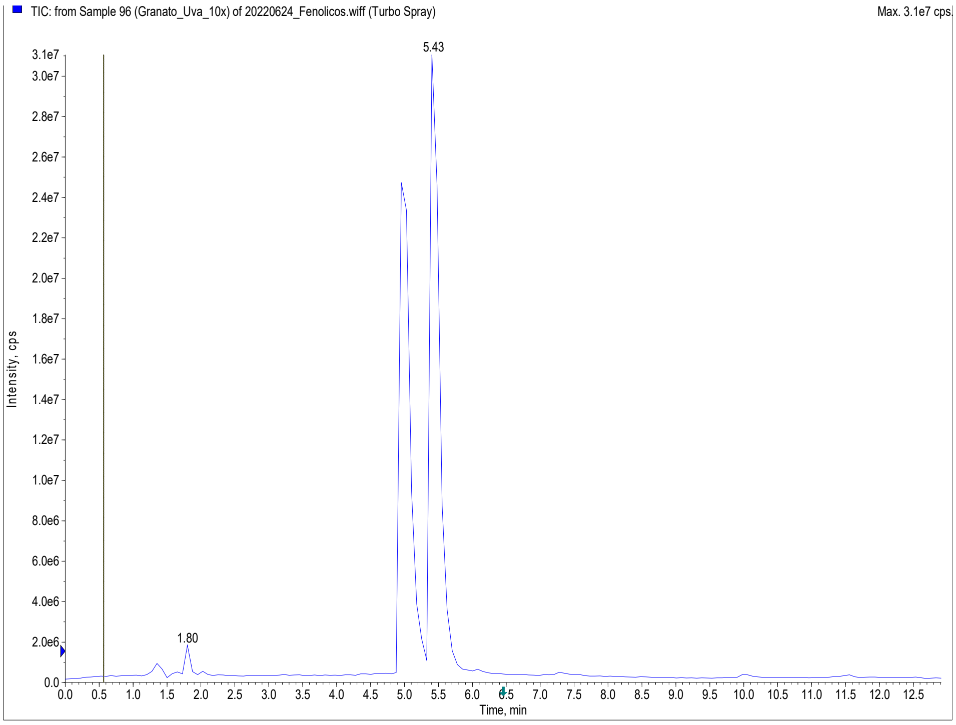
TIC: from Sample 96 (Granato_Uva_10x) of 20220624_Fenolicos.wiff (Turbo Spray)
Max. 3.1e7 cps.
5.43
1.80
Intensity, cps
Time, min

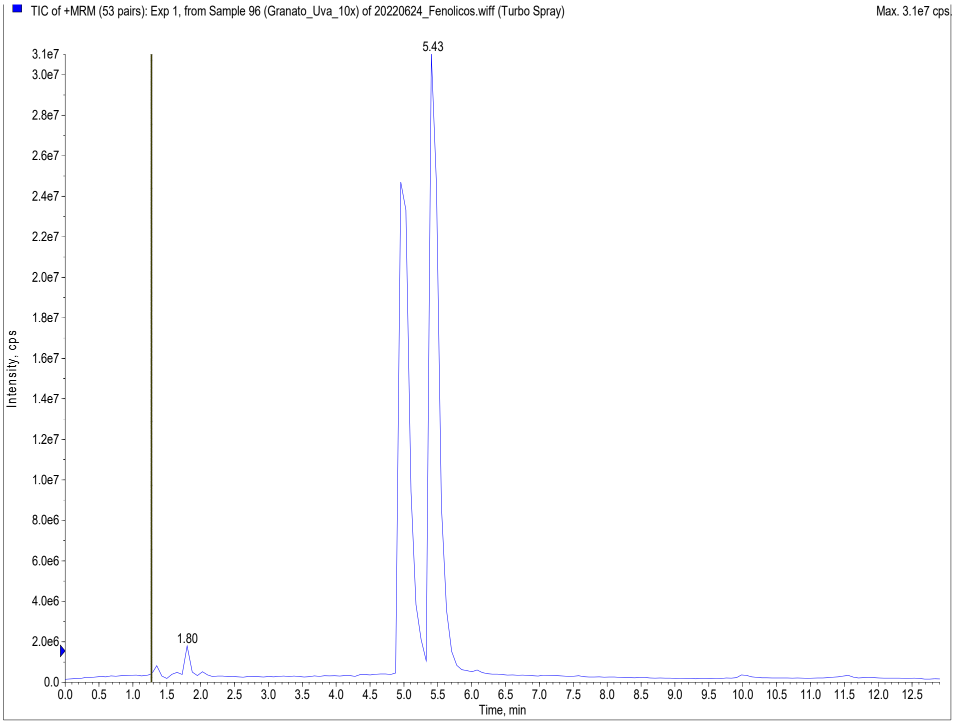
TIC of +MRM (53 pairs): Exp 1, from Sample 96 (Granato_Uva_10x) of 20220624_Fenolicos.wiff (Turbo Spray)
Max. 3.1e7 cps.
5.43
1.80
Intensity, cps
Time, min

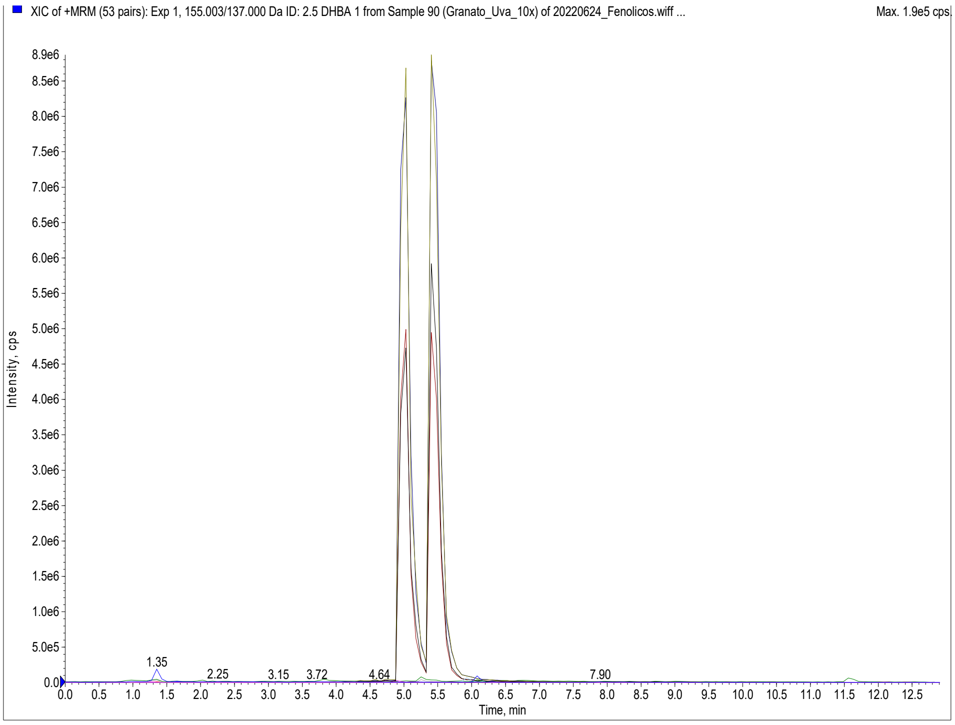
XIC of +MRM (53 pairs): Exp 1, 155.003/137.000 Da ID: 2.5 DHBA 1 from Sample 90 (Granato_Uva_10x) of 20220624_Fenolicos.wiff ...
Max. 1.9e5 cps.
Intensity, cps
Time, min
1.35
2.25
3.15
3.72
4.64
7.90

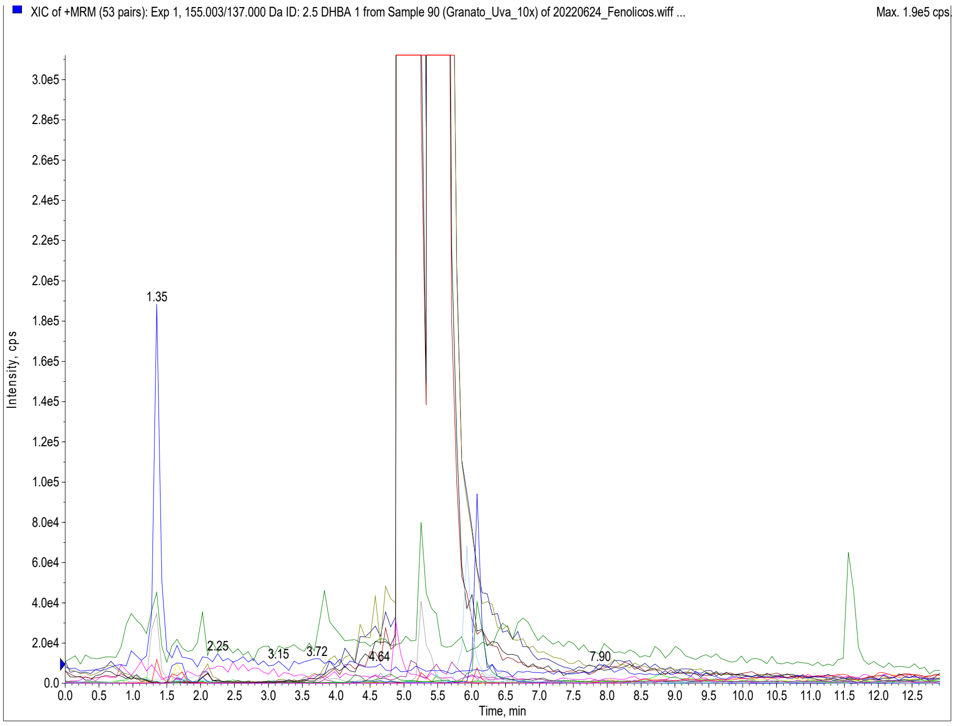
XIC of +MRM (53 pairs): Exp 1, 155.003/137.000 Da ID: 2.5 DHBA 1 from Sample 90 (Granato_Uva_10x) of 20220624_Fenolicos.wiff ...
Max. 1.9e5 cps.
Intensity, cps
Time, min
1.35
2.25
3.15
3.72
4.64
7.90

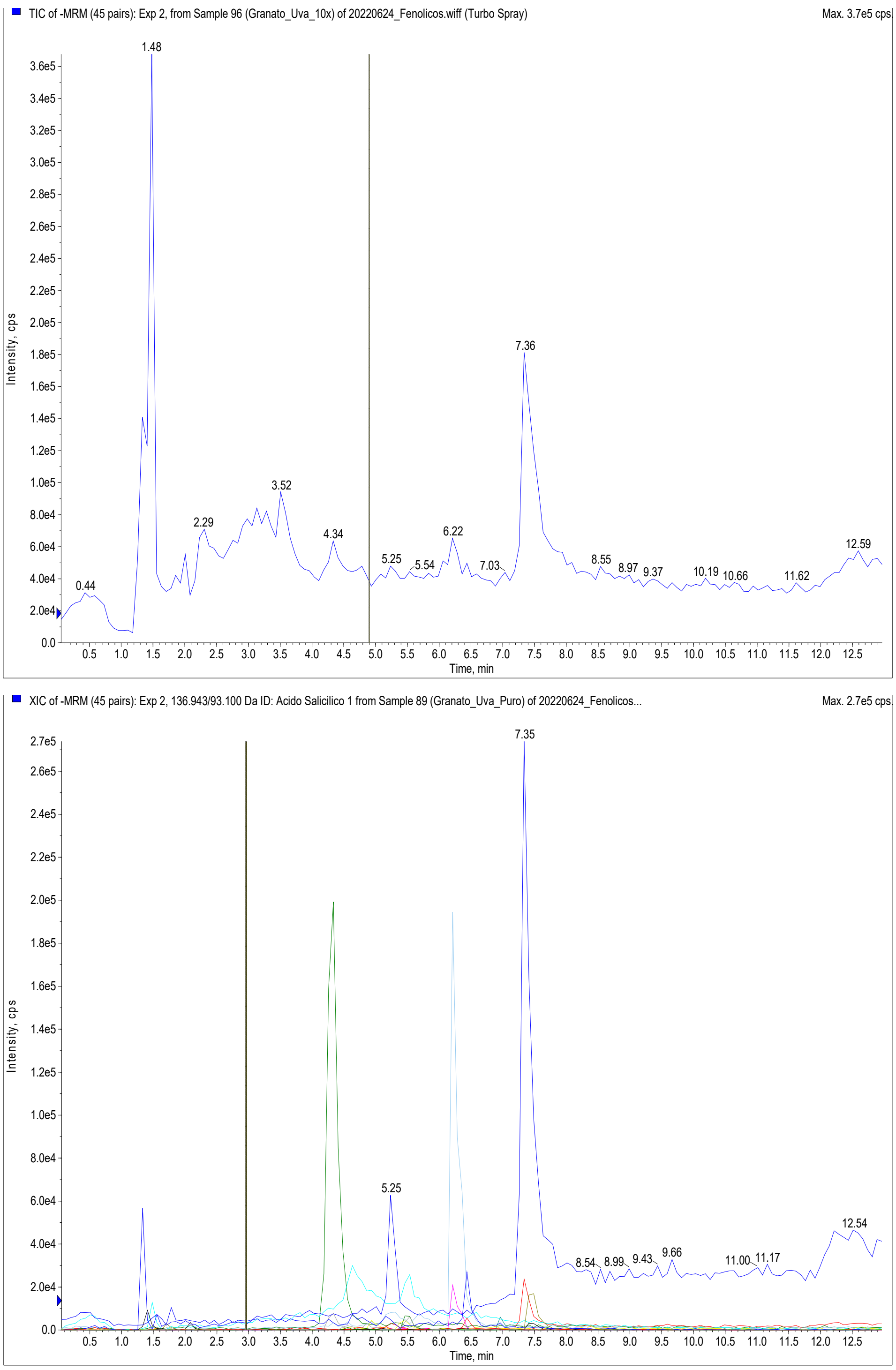

Figure S2. Grape chromatograms.
